# Supplementary material for: The interactome of intact mitochondria by cross-linking mass spectrometry provides evidence for coexisting respiratory supercomplexes
Source: Mol Cell Proteomics. 2017 Dec 8;17(2):216–32. doi: 10.1074/mcp.RA117.000470 (PMC5795388; doi:10.1074/mcp.RA117.000470)
Supplement: Supplemental Data [file supp_RA117.000470_133922_0_supp_23978_fzffwf.zip › spectra_annotation/mito_DR_spectra_annotation/74-1-5-1-5-1.pdf]

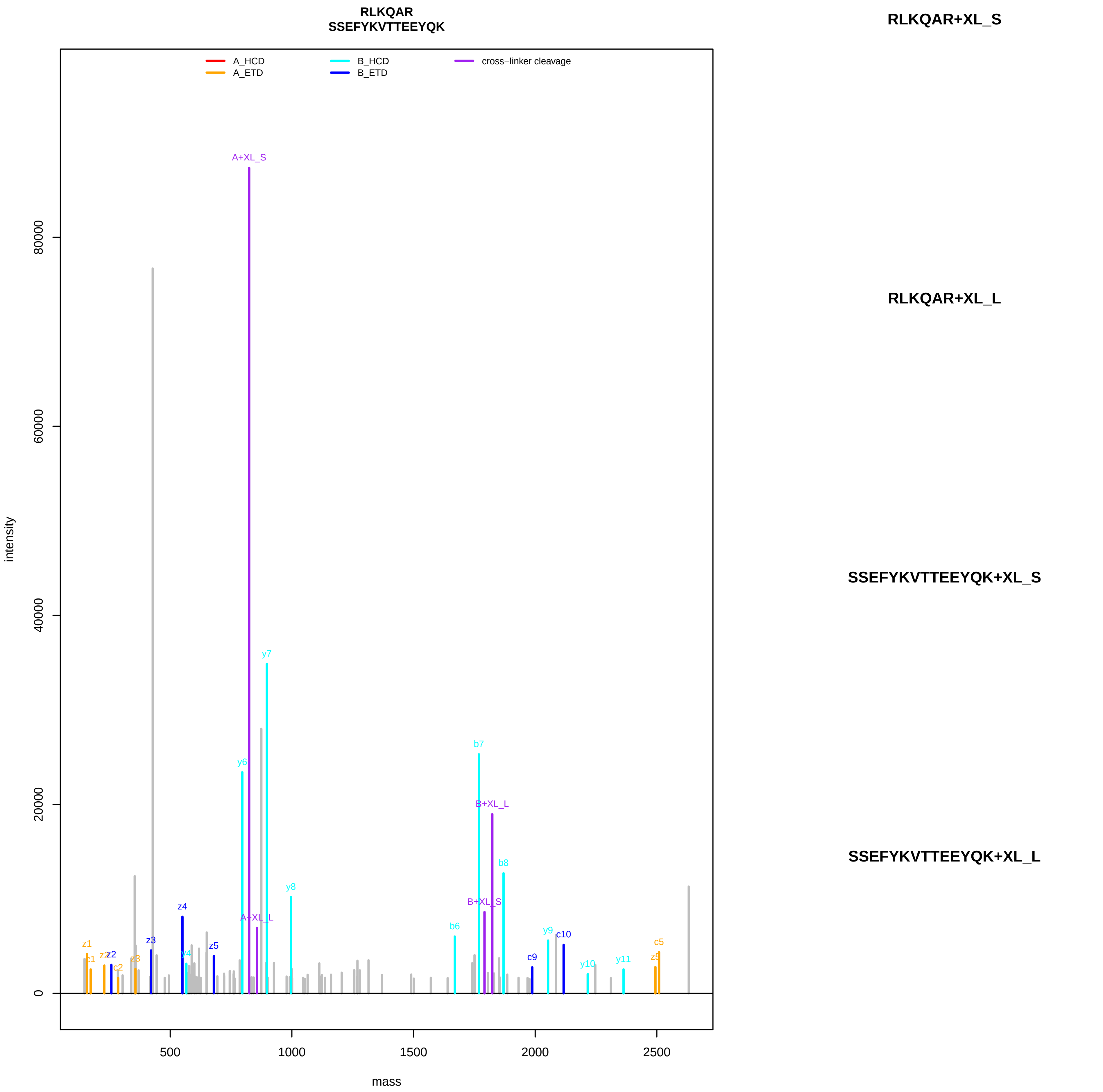
RLKQAR
SSEFYKVTTEEYQK
A_HCD
A_ETD
B_HCD
B_ETD
cross−linker cleavage
A+XL_S
A+XL_L
B+XL_S
B+XL_L
y6
y7
y8
b6
b7
b8
y9
c9
c10
y10
y11
z1
z2
z3
z4
z5
c5
intensity
mass
0
20000
40000
60000
80000
500
1000
1500
2000
2500
RLKQAR+XL_S
RLKQAR+XL_L
SSEFYKVTTEEYQK+XL_S
SSEFYKVTTEEYQK+XL_L
